# Pediatric Palliative Care Patient Transfers of Location at End-of-Life: A 7-Year Retrospective Study

**DOI:** 10.3390/healthcare13202576

**Published:** 2025-10-14

**Authors:** Victoria Sun, Sandra Coombs, Nicole Armitage, Meaghan Dowling, Tiina Jaaniste

**Affiliations:** 1 Department of Palliative Care, Sydney Children’s Hospital, Randwick, NSW 2031, Australia; victoria.sun@student.unsw.edu.au (V.S.); sandra.coombs@health.nsw.gov.au (S.C.); nicole.armitage@health.nsw.gov.au (N.A.); 2 School of Clinical Medicine, University of New South Wales, Kensington, NSW 2052, Australia; 3 Department of Palliative Care, Children’s Hospital at Westmead, Westmead, NSW 2145, Australia; meaghan.starr@health.nsw.gov.au

**Keywords:** medical records, palliative care, patient transfer, pediatrics

## Abstract

**Background/objectives:** Transferring pediatric palliative care patients nearing the end of life (EOL) between care locations is clinically and logistically complex, potentially causing significant stress for families and patients. This study aimed to determine how frequently such transfers occurred in the last two weeks of life, noting the documented reasons for these changes. **Methods:** A retrospective medical record review was conducted for pediatric palliative care patients known to two palliative care services in Sydney, Australia, who died between January 2017 and May 2024. All medical records from the last two weeks of life were reviewed for any transfers that occurred, noting the reasons for these transfers. **Results:** Of 457 patients with available medical records, 44.1% of patients had at least one change in location of care in the last 2 weeks of life: 34.1% had one, 7.0% had two, and 3.1% had three or more changes in location. The most common reason for the final change in location was to receive a higher level of medical care (54.3%), often triggered by a sudden medical event. However, 37.8% of changes involved moving a patient to a less medicalized setting. A smaller proportion of transfers (8.0%) were due to psychosocial reasons. **Conclusions:** To our knowledge, this is the first study to quantify pediatric EOL transfers in the last 2 weeks of life, identifying reasons for these transfers. The findings offer valuable insights to improve planning, reduce unnecessary and potentially stressful transfers, and guide the management of necessary transfers.

## 1. Background

Planned pediatric transfers may occur at the end-of-life (EOL) for a range of clinical, logistical and psychosocial reasons [1,2,3]. Families may reassess their preferences regarding care location as new clinical or psychosocial factors emerge, or as the family’s priorities change [4,5,6,7]. Planned transfers may also occur to help families achieve their preferred location of death. Moreover, transfers sometimes occur when the imminence of death is not recognized [8], prompting hospital admissions in hopes of reversing clinical deterioration.

As a child approaches EOL, some families may desire to move their child from a less medicalized setting, such as home, to hospital, to access greater clinical supports to optimize their child’s comfort. Some parents may prefer the additional supports available at a hospital or hospice so that they can relinquish some of the clinical care roles and to focus on just being with their child [9]. In situations where prognostication is difficult and there is a lack of recognition that a child is dying [10], transfers to hospital may occur with the hope of managing problematic clinical symptoms. It has been documented in the adult EOL context that factors such as acute medical events, uncontrollable symptoms, and the inability to provide necessary care at home may precipitate a patient’s change in location from home to hospital [2]. Similar factors are likely in the pediatric EOL context.

Conversely, some families may choose to move their dying child from hospital to home, desiring a more familiar and less clinical environment, surrounded by loved ones, pets and meaningful belongings [3,11]. For Aboriginal and Torres Strait Islander families, dying on Country, may hold deep spiritual significance enabling greater connection to land and community [12]. Cultural and spiritual motivations for home transfers at EOL have also been documented in a published case series of three pediatric palliative care patients living in diverse communities in the United States [13]. Thus, providing patients and families with the option of transfers to their preferred location at EOL is considered a key aspect of holistic care [14].

Patient transfers close to the EOL are often complex, requiring considerable planning and clinical expertise [13]. This is especially true for children who are dependent on medical technology and require a dedicated critical care transport service to ensure a supported transfer experience with minimal intra-transfer adverse events [6,13,15]. Nevertheless, unplanned transfers, such as those following an ambulance callout, are common; in one Australian study 62% of pediatric palliative care patients (albeit not necessarily in the EOL phase) were transferred to a different care location following an ambulance callout [7]. Reasons for ambulance transfers may include: the family struggling to manage or cope with the child’s care at home [3,16], insufficient community-based services to meet the patient’s and family’s needs [8,17,18], paramedic protocols requiring transfer following administration of certain drugs [19], and paramedic concerns about litigation and the absence or inaccessibility of advance care plans [7,19,20]. Differences in patient transfers at the EOL may reflect variations in service models and service availability across organizations and countries. Nonetheless, international guidance emphasizes the importance of holistic care and supporting family choice in care location [14].

Irrespective of the reasons that motivate a change in patient location in the last days or weeks of life, these transfers can be potentially stressful events for the patient and their family [21]. In light of the medical instability of many patients in the EOL phase, and the difficulty in recognizing when some patients are nearing the EOL [11], clinical complications may occur enroute, potentially even leading to death [22]. Careful planning reduces unnecessary transfers and ensures care needs are met when relocating the patient to the preferred location [20]. In a large Canadian study of adult palliative care patients, it was found that almost half of the patients were transferred to different locations in the last four weeks of life, with 6.3% experiencing five or more transfers [23]. To our knowledge, there is no published data on the frequency and reasons for pediatric palliative care patients being transferred in the final weeks of life.

In light of the lack of data outlining the frequency and reasons for pediatric EOL transfers, the primary aim of this study was to determine how often pediatric palliative care patients were transferred between care locations in the last two weeks of life and the documented reasons for these transfers.

## 2. Methods

### 2.1. Study Design

This study was a multi-centered, retrospective analysis utilizing information from electronic medical records. The study reported in the current manuscript was part of a larger project investigating actual and preferred locations of expected pediatric deaths.

Ethics approval was granted by the Sydney Children’s Hospital Network, Human Research Ethics Committee (2023/ETH02510). Patient and caregiver consent was waived as all data was retrospectively collected without contact with patients, family, or staff.

### 2.2. Cohort

The cohort consisted of pediatric patients who received care from the pediatric palliative care service at either Sydney Children’s Hospital, Randwick or the Children’s Hospital at Westmead, Australia. Inclusion criteria were: (i) patient died before 19 years of age, and (ii) date of death between January 2017 and May 2024. There were no exclusion criteria.

### 2.3. Data Extraction and Coding

Data was extracted from electronic medical records for the two-week period prior to the patient’s death. Key demographic information retrieved for the current study included patient age, sex, primary illness diagnosis and socioeconomic disadvantage. The Index of Relative Socio-economic Disadvantage (IRSD) percentile [24] is an Australian index used to provide information about socioeconomic disadvantage relative to other residential areas based on postcode, with a lower quintile denoting greater disadvantage [24].

Other key information collected from medical records included the frequency, timing, and reasons for patient transfers between locations of care at the EOL, with location change defined as situations in which the patient was moved between different care locations, such as from home to hospital, from hospital to a separate hospice facility, or from one hospital to another hospital, etc. All medical records for the last two weeks of the patient’s death were read and coded by one investigator (VS). A second investigator (TJ) reviewed coding, and any discrepancies or issues were resolved through discussion. Of the 486 patients who met inclusion criteria, 457 had accessible electronic medical records with the potential to code for location over the last 2 weeks of life.

### 2.4. Statistical Analyses

Quantitative analyses were performed using the IBM Statistical Package for the Social Sciences (SPSS Statistics v27.0.1.0). The data elicited was primarily used to carry out descriptive analyses, such as reporting the number of location changes in the last two weeks of life.

Post hoc analyses (*t*-tests) were conducted to assess whether the frequency of patient transfers in the last 2 weeks of life differed in the years where COVID-19 had the greatest impact (2020–2021).

Thematic coding was conducted to analyze the documented reasons for changes in location of care. All data was coded by one researcher (VS) using methods of codebook thematic analysis [25,26]. This process involved developing initial codes based on a review of the literature and data familiarization. Following several readings and trial coding, themes and subthemes were refined until agreement was reached with a second researcher (TJ) that the codebook accurately represented the data.

## 3. Results

### 3.1. Patient and Family Demographics

Patient and family demographic variables for the 457 patients who died over the 7-year study period are presented in Table 1.

### 3.2. Frequency and Timing of Change in Locations of Care

Of the 457 patients with available medical records for the EOL period, 44.1% (*n* = 202) patients had at least one change in location of care in the last 2 weeks of life. Specifically, 34.1% (*n* = 156) had one change in location, 7.0% (*n* = 32) had two changes in location, and 3.1% (*n* = 14) had three or more changes.

The number of patient transfers in the last 2 weeks of life did not differ significantly for deaths occurring during the time of greatest COVID-19 impact in New South Wales (2020–2021) [27]; (*M* = 0.55, *SD* = 0.768) relative to the other years (*M* = 0.66, *SD* = 0.819; *p* = 0.154).

### 3.3. Reasons for Changes in Location of Care

The most commonly documented primary reason for the final change in the patient’s location of care (as seen in Table 2) was to seek a higher level of medical attention (54.3%). This was often triggered by a medical incident, such as a sudden negative change in the patient’s health status. In contrast, 37.8% of changes involved moving a patient away from a more medicalized setting. This typically happened when higher levels of clinical support were no longer necessary or desired, or if the home was the desired location of death. A smaller proportion of transfers (8.0%) were due to psychosocial reasons, for example, if the family found one environment more stressful.

When examining the second last change in patient location, the most common reason why patients transitioned away from a more medicalized setting was because their presenting symptoms had been effectively managed, followed by seeking their preferred location for EOL care.

Post hoc analyses were performed to assess for any differences between patients with 0–1 transfer versus more than 1 transfer in the last 2 weeks of life, with regard to various patient, family and health service factors (See Supplementary Table S1). No significant differences or associations were found.

## 4. Discussion

The ability for patients and their families to move seamlessly between locations is a crucial aspect of EOL care. Pediatric palliative care teams aim to maximize patient comfort, enhance quality of life, and ensure that the family’s care goals are achieved [14,28,29]. However, poor planning may result in frequent and unnecessary transfers, which can add additional stress for patients and their families [21]. To our knowledge, this is the first study to provide data on the frequency and reasons that pediatric palliative care patients change locations at the EOL. This information may enable better planning to occur so that unnecessary transfers may be minimized and necessary transfers optimally managed.

The current study found that 44% of pediatric palliative patients changed location in the last two weeks of life, and that 10% of patients changed location more than once. A recognition of this relatively high prevalence of patient transfers is important, given that it has been documented elsewhere that families of seriously ill children commonly find transfers in care location highly stressful [21,26]. One change in location close to the EOL may help achieve the preferred location of death [13]. Multiple changes may reflect a family’s ability to exercise the flexibility to change between locations. However, it is also possible that multiple changes reflect sub-optimal planning in finding an optimal location, potentially contributing to unnecessary stress on the patient and family [21,30].

The current study found that families desiring a location that can provide a higher level of medical care following a clinical event was found to be the most common reason for a change in location in the last two weeks of life. Similar themes have been reported by Papadatou et al. (2021), who noted that parents may choose a more medicalized environment to enable their child to receive greater clinical support than is possible at home [3]. A medicalized environment may help support symptom management; however, this needs to occur within a broader context of optimizing holistic care for the patient and family. Consideration is needed as to whether the provision of additional community supports would minimize the need for hospital transfers.

A smaller proportion of patients in the current study were transferred with the intention of moving to a less medicalized environment, such as home or hospice. It is possible that these transfers may have been in line with the family’s choice for location of death. Less medicalized environments may be preferred by some families, who believe that the home affords them with greater comfort and flexibility to maintain their parental role [31]. However, in light of increasing treatment options and early phase clinical trials with less predictable outcomes [32], it may be difficult for families and healthcare professionals to recognize when death is nearing. Hence, they may miss the opportunity to arrange a timely transfer to the preferred location of death.

### 4.1. Clinical Directions

Early discussions with the family may enable the clinical team to gain an understanding about their priorities and preferences with regard to preferred EOL care location, so that timely measures may be put in place to help achieve these goals. However, these discussions need regular follow-up, as family preferences may change over [4]. Preparing families in conversations about possible changes in their child’s care needs as they approach death will enable them to make more realistic decisions about EOL locations. Engaging families in these conversations may be challenging, as some families may be reluctant to consider the circumstances leading up to their child’s death, possibly holding on to hope for a cure [33]. Nevertheless, timely discussions regarding preferred locations of EOL care may enable patient transfers to occur in a planned, rather than reactive, way.

Although families may have goals and plans in place regarding preferred EOL care location, the ability to achieve these goals relies on the ability to recognize that the patient may be dying [10]. Careful planning is needed for managing the transfers of all pediatric patients with serious or life-limiting conditions, mindful of the possibility that they may become unstable or experience a sudden deterioration enroute. A summary of issues to consider when planning to move a child who may be close to the EOL between care locations is presented in Table 3.

As shown in Table 3, planned transfers require a thorough understanding by all key individuals of current and potential issues related to symptom management, medication needs, care requirements and equipment requirements. If these are inadequately addressed, patients may be at risk of returning to the hospital [11,34,35], regardless of the preferred location of EOL care. A clear understanding of the family’s wishes and goals of care is also necessary, as well as an awareness of broader psychosocial, cultural and spiritual factors relevant to the patient and their family. These issues are fluid and personnel involved in the transfer require the latest information to ensure that good palliative care is achieved and families can achieve their goals regarding the preferred location of EOL care. Given that patient transfers by definition typically involve multiple teams, there is a need for good communication, document sharing, and coordination between tertiary and local inpatient services, paramedics, community-based services and the family [21,36]. As community palliative care teams primarily care for adult palliative care patients, they may benefit from support from specialist pediatric outreach services [37,38,39].

### 4.2. Limitations

Given that the current study was a retrospective review, the strength of the evidence can only be as good as the level of detail documented in the medical records. Although the current study analyzed data on patients from two tertiary hospitals with a broad patient catchment area, it is not known how generalizable the current results are to other health services and locations. It is possible that some patient transfers were not documented within the medical records, particularly if these transfers did not involve one of the tertiary hospitals from which the study was conducted. This implies that the number of patient transfers documented may have been an underestimate of what actually occurred.

Although post hoc analyses revealed there to be no significant differences in the frequency of EOL pediatric patient transfers in the years most affected by COVID-19 relative to other years, it is not well understood how the decision-making processes regarding patient transfers may have differed for families and health professionals at those times. Anecdotally, challenges were experienced with access to community palliative care services, availability of informal supports to the home environment [40], changes to hospice admission processes, and what may have been considered as “elective” transfers. These factors may have impacted family and healthcare decision-making regarding transfers in unknown ways.

The current study had a good sample size for addressing the key aims of the study; however, it may have been under-powered to examine numerous between-group differences, with some groups being relatively small in number.

### 4.3. Future Research Directions

In light of the frequency with which pediatric palliative care patients are transferred between locations of care when nearing the EOL, it would be valuable for future studies to identify how smoothly these transfers occur and what sorts of challenges arise, especially from the family’s perspective. Moreover, in order to better understand decisions made about changing locations of care for pediatric palliative care patients close to the EOL, it would be useful to know whether families have an awareness of the likely imminence of the child’s death. It is not known to what extent this knowledge would impact on decisions made about the child’s location of care. It may be fruitful to pursue further investigation of between-group differences on a range of patient, family and organizational variables for transfer frequencies with larger samples. Future prospective study designs would also help mitigate some of the current limitations, particularly related to varied documentation practices.

## 5. Conclusions

The current study provides important new data on the relative frequency with which children nearing the EOL are transferred between locations of care. Notably, 44% of patients changed location in the last 2 weeks of life at least once, with 10% having multiple transfers. This highlights the need for careful planning and management to optimize the implementation of necessary transfers and implement strategies to minimize unnecessary transfers. Having the option to change location provides families with greater flexibility to achieve their desired location of care at any particular time. However, numerous changes may occur if there is a lack of understanding and planning for what the families need. Facilitating patient and family preferences regarding location of care, particularly at EOL, is an important aspect of good palliative care. Careful planning and coordination with all key individuals is necessary in order to achieve this goal.

## Figures and Tables

**Table 1:** Demographic characteristics of pediatric palliative patient sample (*N* = 457).

Child Demographics	*n*	%
**Sex**		
Female	212	46.4
Male	245	53.6
**Age at death**		
Infant (0 to <1 year)	102	22.3
Child (1 to <13 years)	244	53.4
Young person (13 to <19 years)	111	24.3
**Primary language at home** (*n* = 456)		
English	361	79.2
Non-English	75	16.4
Both English and non-English	20	4.4
**Primary diagnosis**		
Oncology	207	45.3
Neurology	73	16.0
Cardiology	34	7.4
Metabolic	28	6.1
Respiratory	8	1.8
Genetic	81	17.7
Gastroenterology	17	3.7
Other	9	2.0
**IRSD percentile**		
0–20% (lowest quintile)	118	25.8
21–40%	43	9.4
41–60%	94	20.6
61–80%	85	18.6
81–100%	117	25.6
**Location of death**		
Hospital	230	50.3
Home	125	27.4
Hospice	100	21.9
In transit	2	0.4

**Table 2:** Primary reasons for change in location as documented in medical records.

Primary Reason for Moving	Last Change in Location Before Death (N = 188 *) *n* (%)	Second Last Change in Location Before Death (N = 46) *n* (%)
**Wanting a higher level of medical care (total)**	102 (54.3)	22 (47.8)
Due to sudden medical event	80 (42.6)	16 (34.8)
As planned	22 (11.7)	6 (13.0)
**Wanting to move away from a more medicalized setting (total)**	71 (37.8)	19 (41.3)
Presenting issues managed	16 (8.5)	13 (28.3)
To preferred location for EOL care	55 (29.3)	6 (13.0)
**Psychosocial reasons**	15 (8.0)	5 (10.9)

* It was not possible to identify the reason for all documented transfers. Hence the total numbers of reasons for transfers (*n* = 188) is less than the total number of transfers (*n* = 202).

**Table 3:** Considerations when planning to transfer a pediatric palliative care patient to a different care location when the patient is nearing EOL.

Documentation	Document and make available to all relevant personnel, prior to transfer: Resuscitation status Symptom management plan Advance care plan (including limits to treatments/interventions) Ambulance Plans (if these exist) Goals for EOL
Medication & feeds	Prior to transport, consider: Giving PRN medication for symptom management. Stopping/reducing enteral feeds, approximately 2 h before transport. Check before, during and post-transfer: Knowledge and comfort of paramedics involved with dosage options. Routes of medication administration. Availability of all necessary medications and feeds.
Special equipment & consumables	In the transport vehicle, consider the availability of: Necessary equipment (e.g., oxygen, suction and catheters). Required consumables, including for personal care (e.g., mouth, eyes) and continence. Comfort measures e.g., favorite blanket, music etc. Pressure care, lifting and repositioning options.
Planning for potential clinical deterioration	Plan for the possibility that the patient becomes unstable, deteriorates, or dies enroute, and: Assess the likelihood of such an event and share this information with key individuals. Consider what treatments or interventions would be used. Ensure personnel in transport vehicle are aware of any particular wishes by family. Consider who will verify the death.
Psychosocial, spiritual & community support needs	With respect to the journey, consider: Who will travel together with the patient and how other family members will travel (if applicable). How the parent’s psychosocial needs will be met, particularly if the patient’s condition deteriorates enroute. With respect to the destination location, consider (for patient and family): Availability of psychosocial support, and likely interpersonal dynamics. How well their spiritual and cultural needs can be met. Who will meet the financial costs of the transfer and care at destination.
Communication & coordination between teams	Prior to arrival: If destination is home, ensure home visit has been conducted in a timely way. Inform all key players of a planned transfer (e.g., community teams, local doctor, emergency department alert). Keep local hospital informed in case patient requires subsequent admission. Discussion and planning with other teams/services in case additional transfers are needed if circumstances change, either enroute or from planned destination. Timely involvement of Aboriginal liaison worker if transfer is to help the patient return to Country. Provide detailed handover to personnel assisting with transfer. Advise personnel at destination of departure time and estimated time of arrival. Aim for morning transfer if possible to optimize the opportunity to settle in at the new location. Consider destination access issues (e.g., parking, stairs, pets, and number of family members present at the destination). Consider whether a member of the referring team travels with the transporting team to provide continuity of care. Ensure destination care team has received timely training if necessary to meet patient’s care needs and have support contact numbers as needed. Consider introducing key destination personnel to family before transfer (e.g., by telehealth). On arrival: Provide detailed handover to personnel at destination location. Ensure opportunity for discussion and debrief after the transfer.

## Data Availability

The data presented in this study are available on request from the corresponding author due to privacy and ethical considerations.

## Supplementary Materials

The following supporting information can be downloaded at https://www.mdpi.com/article/10.3390/healthcare13202576/s1: Table S1: Associations (Chi Square Tests and t-tests) Between Number of Changes in Location in Last Two Weeks of life (0 or 1 versus 2 or more) and Patient, Family, and Health Service Factors.

## Abbreviations

The following abbreviations are used in this manuscript:

EOL	End of Life
IRSD	Index of Relative Socio-economic Disadvantage
