# Supplementary material for: Pediatric Palliative Care Patient Transfers of Location at End-of-Life: A 7-Year Retrospective Study
Source: Healthcare (Basel). 2025 Oct 14;13(20):2576. doi: 10.3390/healthcare13202576 (PMC12564485; doi:10.3390/healthcare13202576)
Supplement: Supplementary file 1 [file healthcare-13-02576-s001.zip › healthcare-3846370-supplementary.pdf]

Supplementary Material

Supplementary Table S1: Associations (Chi Square Tests and t-tests) Between Number of Changes of Location in Last Two Weeks of life (0 or 1 versus 2 or more) and Patient, Family, and Health Service Factors

	Number of transports in last 2 weeks			
	0 or 1 (n=?)	2 or more (n=?)	Test statistic	*p* value
Age at death (*N* = 457)	7.33 (SD = 5.99)	8.20 (SD = 6.08)	$t(55.2) = -.916$	.926
Identified to be at terminal phase				
Yes	347 (89.7%)	40 (10.3%)	x^2=(1, N = 451) = .055	.814
No	58 (90.6%)	6 (9.4%)		
IRSD percentile (*N* = 457)	52.7 (SD=32.5)	42.6 (SD=33.4)	$t(55.0) = 1.95$	.853
Timeliness of PPC referral				
Timely	311 (90.4%)	33 (9.6%)	x^2=(1, N = 393) = .018	.892
Untimely or unsure	44 (89.8%)	5 (10.2%)		
Home visit				
Carried out	174 (85.3%)	30 (14.7%)	x^2=(1, N = 431) = 6.61	.010
Not carried out	211 (93.0%)	16 (7.0%)		

Note. As there were 5 statistical tests, the Bonferroni-adjusted *p* value needed for significance is .01.
